# CAREGIVERS’ INTERACTIONS WITH HEALTH CARE PROVIDERS AND THEIR WELL-BEING BY RACE AND ETHNICITY AND GENERATION

**DOI:** 10.1093/geroni/igad104.2643

**Published:** 2023-12-21

**Authors:** Heehyul Moon, Sunshine Rote, Sol Baik

**Affiliations:** University of Louisville, Louisville, Kentucky, United States; University of Louisville, Louisville, Kentucky, United States; University of Virginia, Charlottesville, Virginia, United States

## Abstract

Black and Hispanic family caregivers often face higher levels of care demands than White caregivers, due to factors such as limited resources, cultural differences, and systemic and structural inequalities. Family caregivers from different generations (Baby Boomer vs. Generation X & Millennial ) may approach and experience caregiving process and its health effects to a different extent. Caregivers often interact with healthcare providers on behalf of their care recipients, and these experiences can vary based on caregiver background (race and ethnicity and generation). The current study aimed to explore these interactions and their impact on caregivers’ well-being using nationally representative data from the National Study of Caregiving (NSOC, round 11 (N=1,455; 418 Black caregivers, 926 Non-Hispanic White caregivers, and 111 Hispanic caregivers)). Most caregivers were adult children (70%) and female (70%), with approximately 68% were from the baby boomer generation. While Black and Hispanic caregivers were significantly less likely than White caregivers to communicate with medical providers (χ2(df=2)=12, p<.001), baby boomer caregivers provided more assistance with speaking to medical providers than caregivers from younger generations (χ2(df=1)=4, p<.05). Our regression results indicate that Hispanic caregivers who had more frequent communication with medical professionals reported more depressive symptoms and poorer health, while Black caregivers who rarely communicated with providers reported worse outcomes. Future research should examine the nature and quality of these interactions with healthcare providers to improve caregivers’ well-being.

